# Poncet's Disease Initially Managed as Osteoarthritis: Case Report From a Peripheral Centre in Kenya

**DOI:** 10.1002/ccr3.71280

**Published:** 2025-10-14

**Authors:** Irene Biomdo, Paul Bundi Karau

**Affiliations:** ^1^ Chuka County Referral Hospital Chuka Kenya; ^2^ Kenya Methodist University & Meru Teaching and Referral Hospital Meru Kenya

**Keywords:** constitutional symptoms, endemic, polyarthritis, Poncet's disease, tuberculosis

## Abstract

Tuberculosis (TB) remains a major public health challenge in Kenya. Though a multisystemic disease, some of the manifestations of tuberculosis such as Poncet's disease are rare and should be considered in patients presenting with symmetric polyarthritis in TB‐endemic areas. We present a case of a 49‐year‐old male patient who presented with sub‐acute ankle and foot swelling and was initially managed for osteoarthritis without improvement. At initial presentation, the history of constitutional features was equivocal, missing a potential opportunity to diagnose a systemic illness. On developing constitutional features of weight loss and fevers, a suspicion of tuberculosis was made, and a diagnosis of pulmonary tuberculosis confirmed on chest imaging and sputum Gene Xpert test. This case highlights the importance of maintaining a high index of suspicion when dealing with patients with polyarthritis.

**Summary**

- Patients presenting with arthritis and constitutional features of fevers and weight loss in high burden TB areas should be screened for Poncet's disease.

## 1 Introduction

Kenya is a high‐burden TB country and ranks among the 30 countries which account for over 80% of all TB cases worldwide [1]. It is estimated that TB affects 267 people for every 100,000 population in Kenya, yet up to 40% of the affected individuals remain undetected and untreated [1].

Poncet's disease, a joint manifestation of active tuberculous infection, presents as a non‐erosive inflammatory arthritis [2], and mostly affects the ankle joint; although other joints, like knees, shoulders, elbows, wrists, metacarpophalangeal, and metatarsophalangeal joints, can be affected [3].

In a region with a high TB burden, the clinician must have a high index of suspicion not to miss this potentially treatable condition, especially among patients presenting with inflammatory, non‐erosive, non‐deforming arthritis. We hereby present a case of Poncet's disease managed in a peripheral centre in Kenya, which was initially diagnosed as osteoarthritis.

## 2 Case Report

### 2.1 Case History

A 49‐year‐old man presented to the Orthopedic clinic with a history of bilateral ankle and foot swelling for three weeks. He had difficulty walking due to pain but did not experience any morning stiffness of the joints. He reported no history of trauma and had no other affection of his large or small joints. Foot and ankle x‐rays showed periarticular osteoporosis suggestive of early inflammatory arthritis of the feet. There were no erosions or joint space narrowing identified. Initial blood workups were unremarkable with a non‐reactive Rheumatoid Factor test. The patient was managed for ankle osteoarthritis, and on subsequent follow‐up visits, he showed no improvement apart from occasional symptomatic pain relief. Of note was that he was unable to resume his work as a mechanic. He was referred to the Medical outpatient clinic (MOPC) due to generalized body weakness and loss of weight, where he revealed that he had loss of appetite, malaise, and lethargy. He had no vomiting, no abdominal discomfort, and no change in bowel habits. He still had the ankle and foot swelling with difficulty ambulating due to pain. He reported no cough, orthopnoea, or paroxysmal nocturnal dyspnoea. He didn't have any fever or night sweats but had difficulty sleeping well at night. No symptoms suggestive of a sexually transmitted illness were elicited.

### 2.2 Examination Findings

On general examination, the patient was noted to have signs of wasting, which were hair thinning, zygomatic bone prominence, weighed 45 kg, and height 170 cm. He had mild pallor, no jaundice, or lymphadenopathy. Oxygen saturations were 98% on room air.

Both feet were still swollen up to the level of the ankle joints with non‐pitting oedema; tenderness was elicited on his ankle joints and his metatarsophalangeal and intertarsal joints, which were inflamed. Respiratory examination revealed a normal respiratory rate of 15; however, there were reduced breath sounds in both upper lung zones.

Cardiovascular examination was unremarkable apart from tachycardia of 116 beats per minute. Abdominal examination revealed tipped splenomegaly.

### 2.3 Investigations

A complete blood count showed normal white blood cell counts of 4 × 10^3^ with lymphopenia, mild anemia of 10 g/dL with a normocytic normochromic picture (Mean corpuscular volume 84 fl), and relatively low platelets at 102 × 10^9^.

The erythrocyte sedimentation rate (ESR) of 28 mm/h, rapid malaria antigen test was negative, Human Immunodeficiency Virus‐1 (HIV‐1) antibody test was negative. The Anti‐Streptolysin O Titre was qualitatively positive. The urea was elevated 20 mmol/L, creatinine at 171 μmol/L, mild hyponatremia of 132 mmol/L, normokalemia of 4 mmol/L, and normal chloride of 94 mmol/L.

**FIGURE 1:**
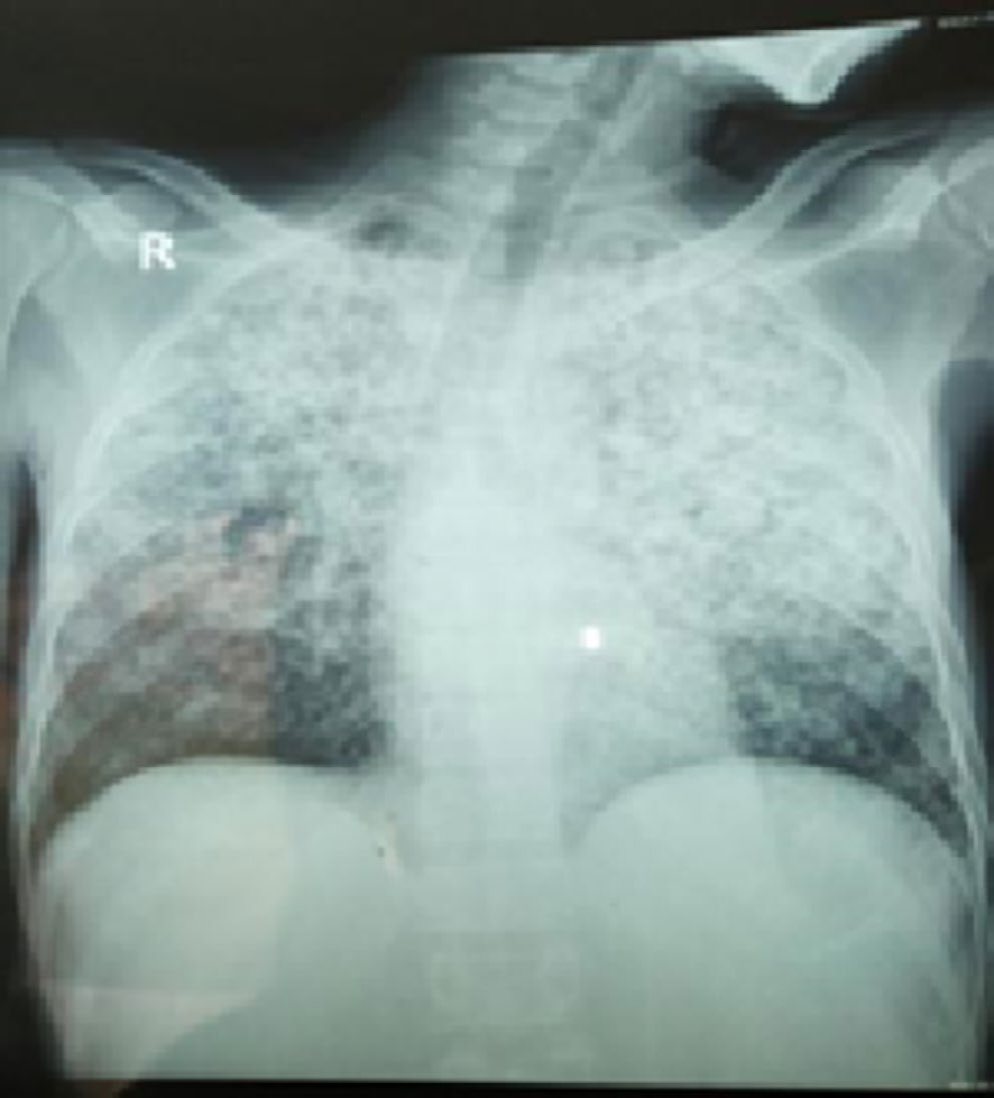
Postero‐anterior chest radiograph showing military‐like opacities on both lungs with relative sparing of lung bases.

**FIGURE 2:**
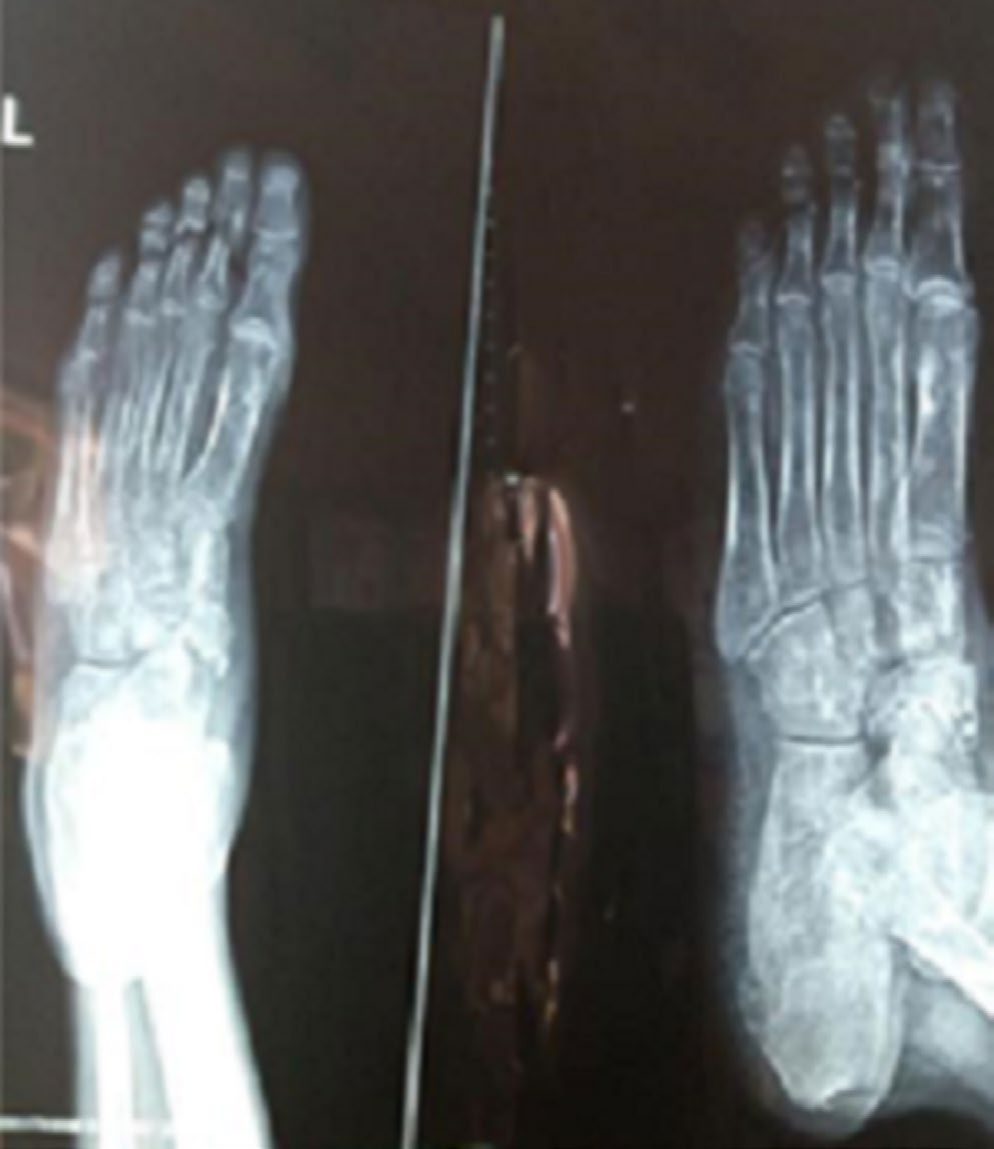
Foot x‐ray radiograph showing non‐erosive, non‐deforming arthritis of the ankle and mid‐foot joints.

Due to these constitutional features which developed while he was on follow‐up, investigations for tuberculosis were undertaken. A chest radiograph revealed bilateral upper lobe dense opacifications sparing the basal area (Figure 1) and a CT scan of the chest confirmed the features of pulmonary tuberculosis in a background of bronchiectasis, where there were bilateral diffuse pulmonary nodules with extensive bilateral tree‐in‐bud appearance predominantly at the upper lobes. A plain radiograph of the ankle and foot showed non‐erosive arthritic changes (Figure 2).

A positive sputum Gene Xpert test further confirmed the diagnosis with Rifampicin resistance detected. The patient was commenced on the 18‐month regimen of Multi‐drug resistant Tuberculosis, and within weeks of starting treatment, the arthritis on his ankle and feet had resolved.

## 3 Discussion

Poncet's disease is a sterile reactive arthritis that occurs during active tuberculous infection without direct mycobacterial presence in the involved joints. This polyarthritis is a non‐erosive, non‐destructive inflammatory process where no other cause can be detected [2, 4].

The condition is different from the widely known TB monoarthritis where there is demonstration of mycobacterial infection in a joint with associated erosive and destructive changes [2]. It also differs from Reactive arthritis in that the duration of the arthritis is much longer, has partial relief from inflammatory drugs but with complete resolution within a few weeks of commencement of antitubercular drugs [5].

PD is relatively uncommon, with 35% of the case reports published coming from India [6]. The burden of tuberculosis is enormous, accounting for 1.13 million deaths among HIV‐negative individuals globally in 2022, with most of the estimated deaths occurring in South East Asia at 53% and 27% in Africa [7].

Previously, PD was thought to be a diagnosis of exclusion up until recently when numerous global case reports and series have made it a recognized entity with proposed diagnostic criteria. In a systematic study of 198 cases and reports, the most common site of active TB infection in PD was in the lungs (43.2%), while other extrapulmonary sites such as lymphatic, renal, bone, and skin made up the rest [6].

The most common rheumatological manifestation of PD is oligoarthritis at 40%, followed by polyarthritis (27.6%) and monoarthritis (24.6%). The most frequently affected joints were the ankles (63%) and knees (59%) while the shoulders, elbows, wrists, metacarpophalangeal, metatarsophalangeal, and interphalangeal joints were less involved [6, 8]. A recent case series [3], showed that the most common joint affection in PD was in the ankles followed by the knee. They proposed a diagnostic criterion where a case was termed as definite PD if there was a non‐erosive, non‐deforming inflammatory arthritis with concurrent diagnosis of extra‐articular tuberculosis and the joint affection showed complete response to antitubercular therapy. The limitation of this criterion would be that a patient may not fulfill all the parameters required at presentation.

While this condition has been hypothesized to be an immunologically mediated disease caused by molecular mimicry between TB antigens and host cartilage in some individuals [8] others authorities believe that it's a hypersensitive immune cell‐mediated response to the tuberculoprotein, resulting in an inflammatory reaction in the joint spaces [9, 10]. The term tuberculous rheumatism was coined due to the observation that the joint involvement improved spontaneously weeks after anti‐TB therapy was completed without sequelae.

PD differs from other seronegative arthritides in that it has a predilection for the lower limbs presenting as an oligo or polyarthritis; it has no chronicity of symptoms and lacks axial involvement [2, 11].

Unless a high index of suspicion and proper follow‐up is undertaken, a number of patients with Poncet's disease would be missed or diagnosed late in the course of the disease. Our patient was on follow‐up for osteoarthritis without improvement. This should have prompted the initial physician to look for other causes. Being in a TB endemic area, Poncet's disease must always be considered as a potential cause of arthritis in patients unresponsive to normal arthritis medication and those with new onset constitutional features such as fevers, night sweats, and weight loss.

Our patient had polyarthritis affecting his ankle joints and metatarsophalangeal joints bilaterally, with no other joint involvement. He had no evidence of spondyloarthritis. Comparably, the typical TB monoarthritis, which is an active tubercular infection of the joint, has a predilection for the weight‐bearing joints such as the hip and knee, with associated joint destruction [12]. Synovial fluid aspirate in such cases demonstrates the presence of Mycobacteria tuberculosis, and as the name suggests, it is usually monoarticular [13].

Our patient's imaging of the foot was unremarkable for any erosions or destructive joint changes despite the symptoms being present for more than six weeks. Gonococcal arthritis usually has a migratory nature with associated papules and pustules elsewhere [10], and this was ruled out in our patient. Another differential is concomitant palindromic rheumatoid arthritis, but this typically has less than six weeks of symptoms and responds well to NSAIDS [14].

The patient in our report had only partial relief from anti‐inflammatory drugs given. Lofgren syndrome is an important differential diagnosis that can mimic PD as it is characterized by bilateral ankle involvement with hilar lymphadenopathy and erythema nodosum [15]. The chest imaging of our patient showed features of active tuberculosis in the upper lobes and no hilar adenopathy. Finally, our patient's bilateral ankle arthritis resolved completely after a few weeks of initiation of Anti‐TBS, which is usually the case with PD.

## 4 Conclusion

PD is not commonly diagnosed in Sub‐Saharan Africa and can delay the initiation of life‐saving anti‐tuberculous drugs. The patient in our report was initially managed as a case of osteoarthritis and was on NSAIDs and joint supplements for close to two months before the actual diagnosis was made. This prolonged phase of untreated active TB may have contributed to rifampicin‐resistant TB. We believe that the unusual presentation of bilateral foot and ankle arthritis, the lack of a detailed history, and the failure to perform a full physical examination led to the delay in diagnosis. A high clinical suspicion and good clinical acumen should be applied in cases of patients presenting with unresolved arthritis on conventional treatment. In such cases, more imaging of the chest and abdomen is warranted. A good collaboration between the orthopedic surgeons, rheumatologist, and chest physicians is the much‐needed multidisciplinary approach for good outcomes.

## Author Contributions

**Paul Bundi Karau:** conceptualization, data curation, formal analysis, methodology, validation, writing – original draft, writing – review and editing. **Irene Biomdo:** conceptualization, data curation, formal analysis, investigation, methodology, writing – original draft, writing – review and editing.

## Consent

Written informed consent was obtained from the patient to publish this report in accordance with the journal's patient consent policy.

## Conflicts of Interest

The authors declare no conflicts of interest.

## Data Availability

The data that support the findings of this study are available in this article. Further enquiries can be directed to the corresponding author.
